# Supplementary material for: Probing Notch1-Dll4 signaling in regulating osteogenic differentiation of human mesenchymal stem cells using single cell nanobiosensor
Source: Sci Rep. 2022 Jun 20;12:10315. doi: 10.1038/s41598-022-14437-x (PMC9209437; doi:10.1038/s41598-022-14437-x)
Supplement: Supplementary file 1 — Supplementary Information. [file 41598_2022_14437_MOESM1_ESM.docx]

**Probing Notch1-Dll4 Signaling in Regulating Osteogenic Differentiation of**

**Human Mesenchymal Stem Cells using Single Cell Nanobiosensor**

Yuwen Zhao^1, 2^, Rui Yang^1, 3^, Zoe Bousraou^1^, Kiarra Richardson^1,4^, Shue Wang^1,*^

^1^Department of Chemistry, Chemical and Biomedical Engineering, University of New Haven, West Haven, CT, 06516, USA

^2^Department of Biomedical Engineering, Lehigh University, Bethlehem, PA, 18012, USA

^3^Department of Biomedical Engineering, University of Connecticut, UConn Health, Farmington, CT, 06030, USA

^4^Department of Biomedical Engineering, Duke University, Durham, NC, 27708, USA

Corresponding author: Dr. Shue Wang (e-mail: swang@newhaven.edu)

**Fig. S1.** Random probe expression tracking of hMSCs during osteogenesis for 7 days **.**

**Fig. S2.** Dll4 mRNA expression tracking of hMSCs during osteogenesis for 7 days..

**Fig. S3.** Representative bright field and fluorescence images of hMSCs after 5 days of osteogenic induction.

**Fig. S4.** Representative bright field and fluorescence images of hMSCs after 5 days of osteogenic differentiation under different treatments.

**Fig. S5.** Schematic illustration of hMSCs 3D spheroid formation.

**Fig. S6**. Comparison of Dll4 mRNA expression of hMSCs in 3D spheroids.

**Fig. S7.** Illustration of Notch signaling.

**Tab. S1.** LNA/DNA probes and quencher sequences


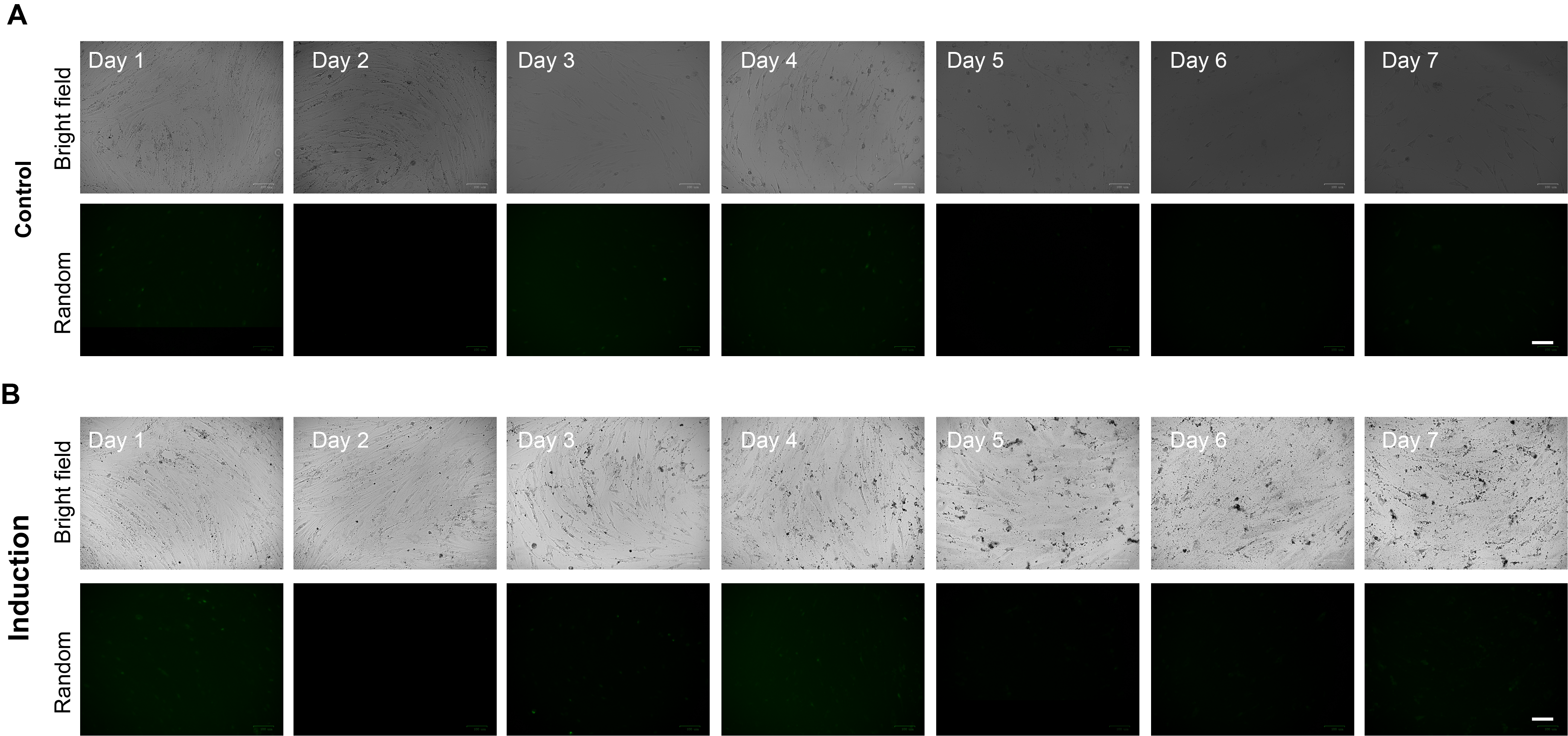


**Fig. S1**. Random probe expression tracking of hMSCs during osteogenesis for 7 days. hMSCs were cultured in basal medium **(A)** and osteogenic induction medium **(B)** for 7 days, respectively. Green: random probe. Scale bar: 100 μm.


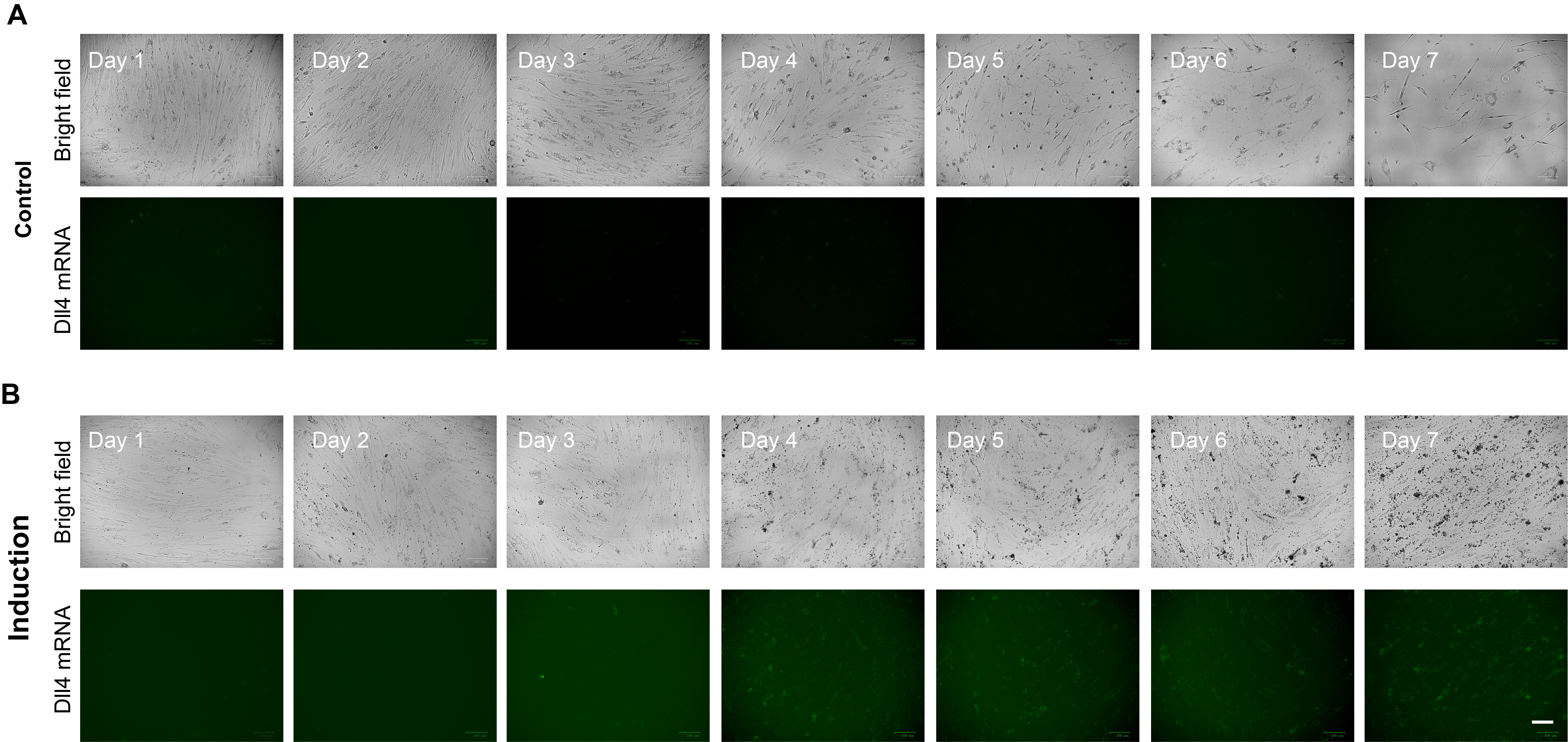


**Fig. S2.** Dll4 mRNA expression tracking of hMSCs during osteogenesis for 7 days.  **(A)** Representative bright field and fluorescence images of hMSCS cultured in basal medium. **(B)** Representative bright field and fluorescence images of hMSCS cultured in osteogenic induction medium. Green: Dll4 mRNA. Scale bar: 100 μm.


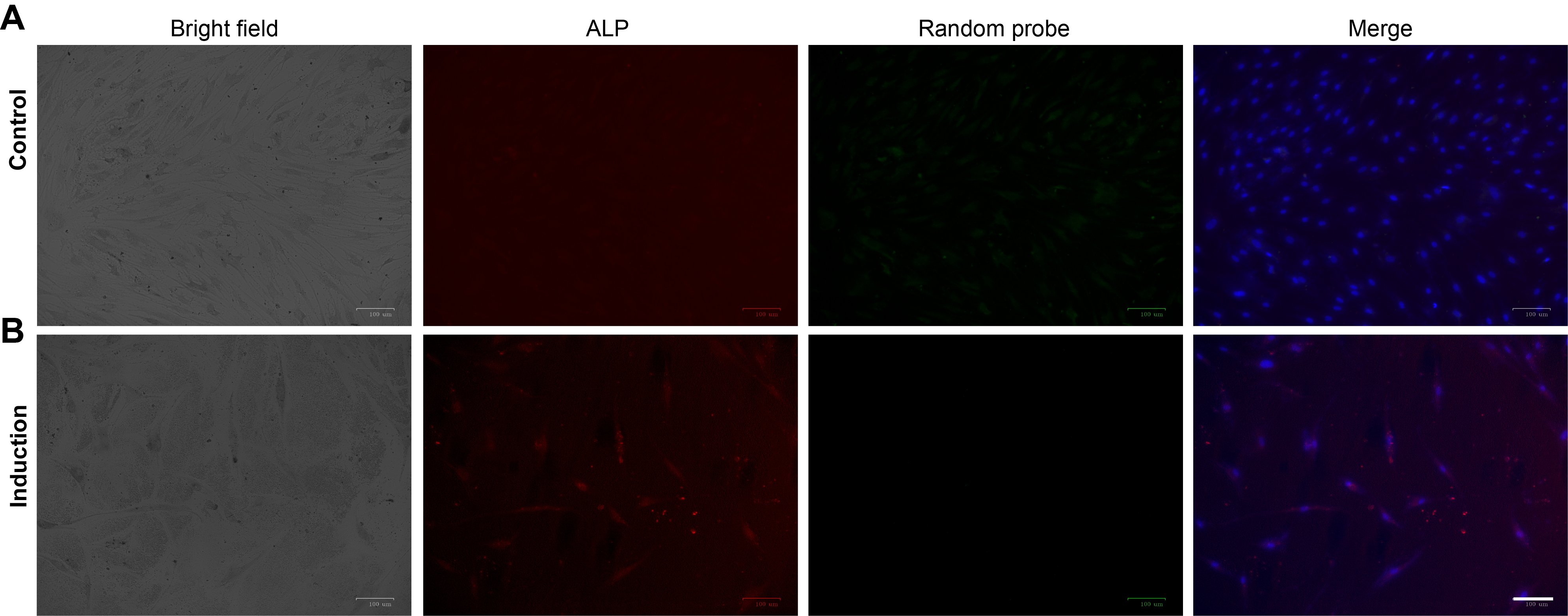


**Fig. S3**. Representative bright field and fluorescence images of hMSCs after 5 days of osteogenic induction. hMSCs were transfected with a random probe and stained with ALP and Hoechst 33342. **(A)** Representative images of hMSCs cultured in basal medium. **(B)** Representative images of hMSCs cultured in osteogenic induction medium. Green: random probe; red: ALP; blue: Nucleus. Scale bar: 100 μm


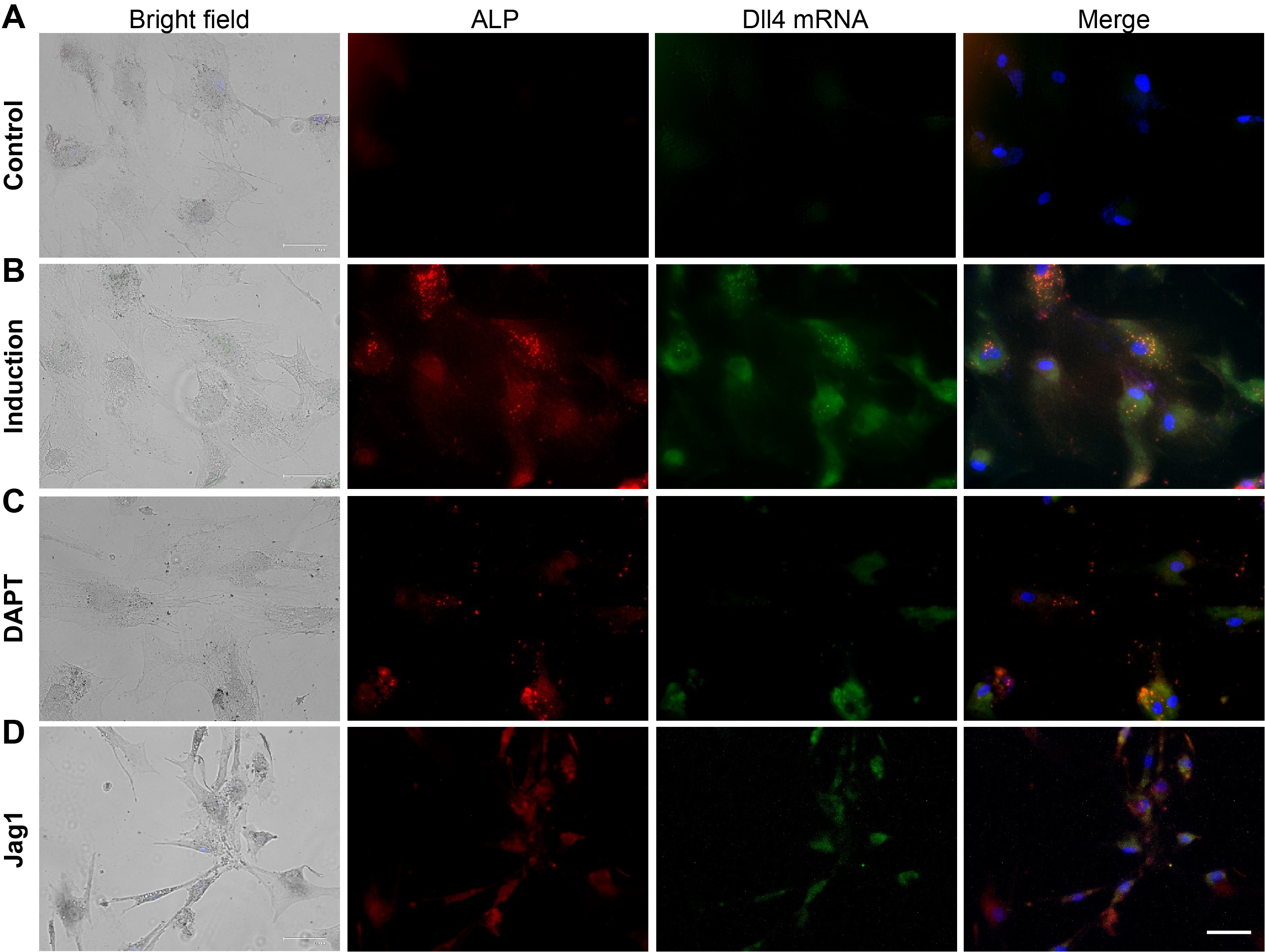


**Fig. S4**. Representative bright field and fluorescence images of hMSCs after 5 days of osteogenic differentiation under different treatments. **(A)** Control group; hMSCs were cultured in the basal medium without treatments.  **(B)** Induction group; hMSCs were cultured in osteogenic induction medium without treatments. **(C)** DAPT group, hMSCs were cultured in induction medium and treated with DAPT at a concentration of 20 μM for 5 days. **(D)** Jag1 group, hMSCs were cultured in induction medium and treated with Jag1 peptide at the concentration of 40 μM for 5 days. Green: Dll4 mRNA; red: ALP; blue: Nucleus. Scale bar: 100 μm.


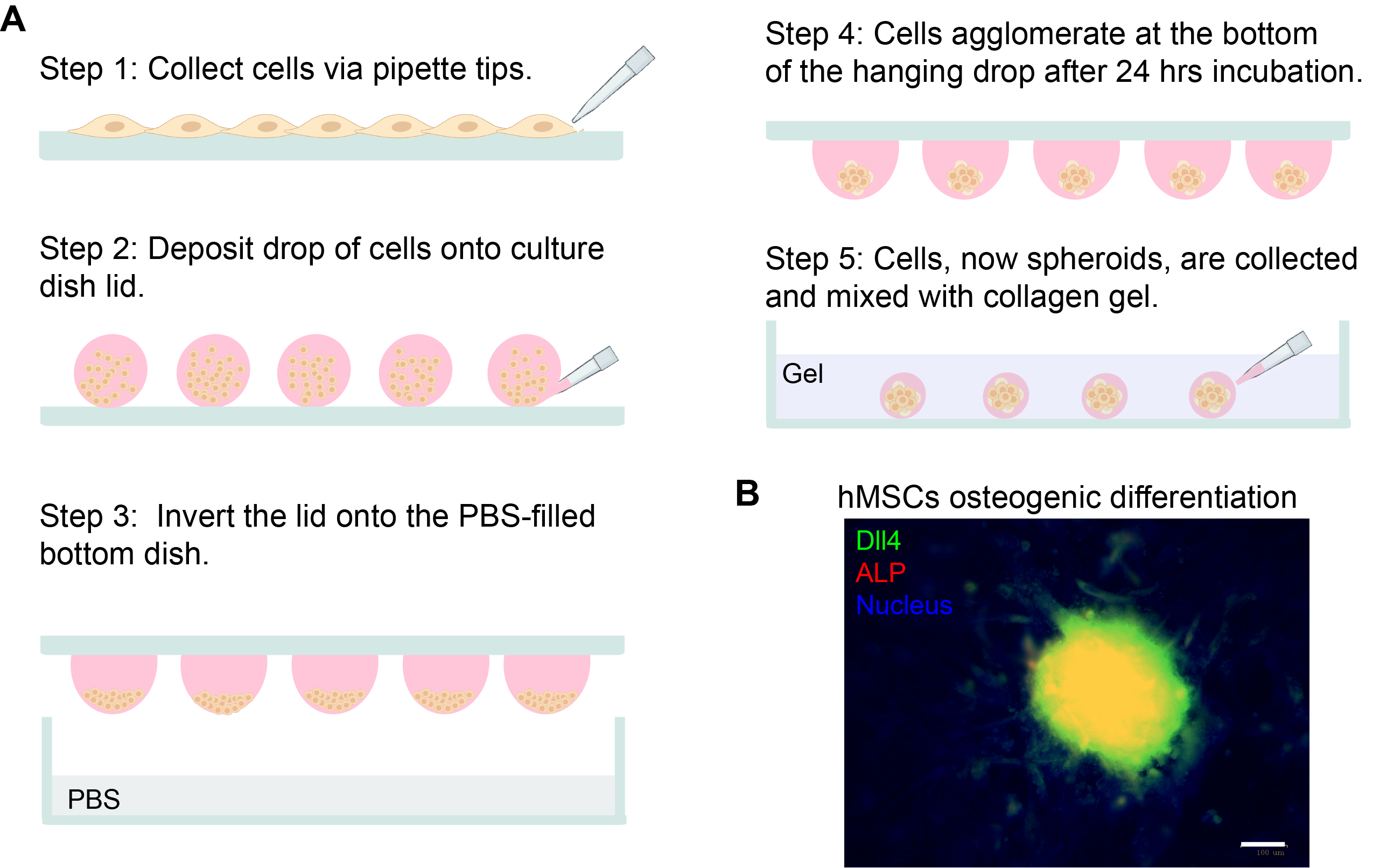


**Fig. S5**. Schematic illustration of hMSCs 3D spheroid formation. **(A)** Process flow of spheroid formation using a hanging drop method. Cells were harvested and seeded at a concentration of 8x10^3^ cells/drop on a culture dish lid. Spheroids were then incubated as hanging drops for 72 hrs, and then transferred into the Matrigel. **(B)** Representative images of 3D spheroids after 10 days of osteogenic differentiation. Green: Dll4 mRNA; red: ALP; blue: Nucleus. Scale bar: 100 μm.


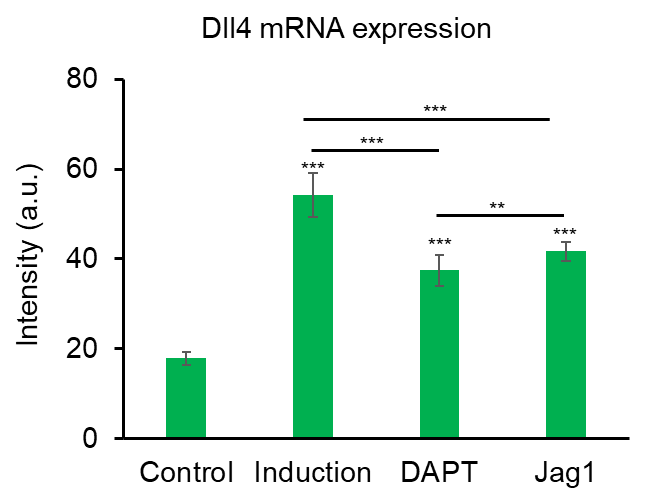


**Fig. S6**. Comparison of Dll4 mRNA expression of hMSCs in 3D spheroids. Mean fluorescence intensity of Dll4 in hMSCs spheroids were measured and quantified under different treatments. Data represent over 50 spheroids in each group and are expressed as mean± s.e.m. (n=4, ***, *p*<0.001, **, *p*<0.01)

**
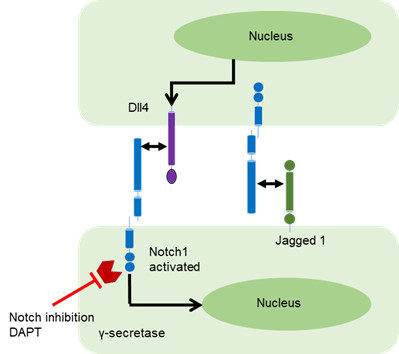
**

**Fig. S7.** Illustration of Notch signaling.

**Tab. S1.** LNA/DNA probes and quencher sequences

| Name | | Sequence (5’-3’) | Fluorophore |
| --- | --- | --- | --- |
| Dll4 mRNA | Donor | +AA +GG +GC +AG +TT +GG +AG +AG +GG +TT | /56-FAM |
|  | Quencher | +TT +CC +CG +TC +AA | /3-Iowa BlackFQ |
|  | Target | AA CC CT CT CC AA CT GC CC TT |  |
| Random | Donor | +AC+GC+GA+CA+AG+CG+CA+CC+GA+TA | /56-FAM |
|  | Quencher | +TG +CG +CT +GT +TC | /3-Iowa BlackFQ |
|  | Target | TA TC GG TG CG CT TG TC GC GT |  |

* + represents LNA monomer
